# Unveiling the Nature of lignin’s Interaction with Molecules: A Mechanistic Understanding of Adsorption of Methylene Blue Dye

**DOI:** 10.1021/acs.biomac.4c00371

**Published:** 2024-06-17

**Authors:** Oleg Tkachenko, Daryna Diment, Davide Rigo, Maria Stro̷mme, Tetyana M. Budnyak

**Affiliations:** † Division of Nanotechnology and Functional Materials, Department of Materials Science and Engineering, Uppsala University, Uppsala 751 05, Sweden; ‡ Department of Bioproducts and Biosystems, Aalto University, Espoo 02150, Finland; § Department of Earth Sciences, Uppsala University, Uppsala 751 05, Sweden; ∥ Wallenberg Initiative Materials Science for Sustainability, Department of Earth Sciences, Uppsala University, Uppsala 751 05, Sweden

## Abstract

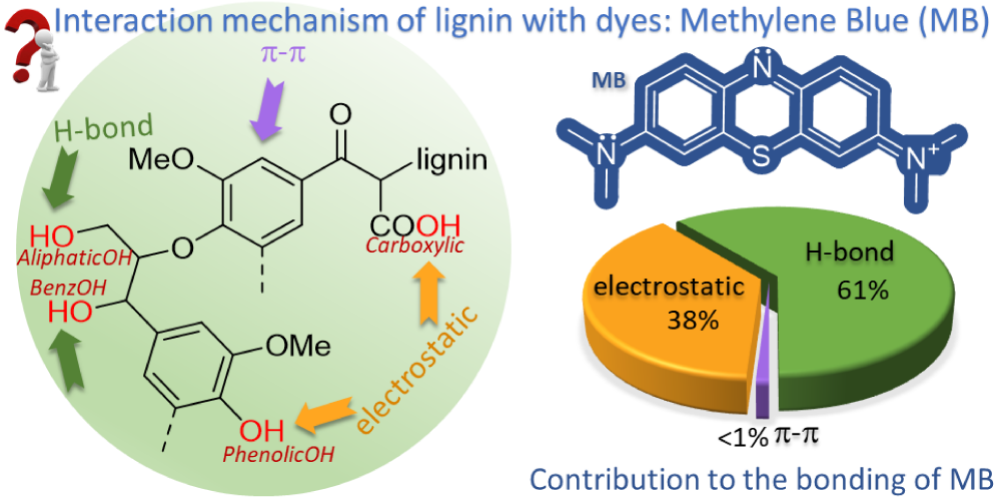

The valorization of lignin into advanced materials for water and soil remediation is experiencing a surge in demand. However, it is imperative that material research and manufacturing be sustainable to prevent exacerbating environmental issues. Meeting these requirements necessitates a deeper understanding of the role of lignin’s functional groups in attracting targeted species. This research delves into the interaction mechanisms between lignin and organic molecules, using the adsorption of the cationic dye Methylene Blue (MB^+^) as a case study. Herein, we aim to quantitatively estimate the contribution of different interaction types to the overall adsorption process. While carbonyl groups were found to have no significant role in attraction, carboxylic groups (−COOH) exhibited significantly lower adsorption compared with hydroxyl groups (−OH). Through alternately blocking aliphatic and phenolic −OH groups, we determined that 61% of the adsorption occurred through hydrogen bonding and 38% via electrostatic interactions. Performance studies of modified lignin along with spectroscopic methods (XPS, FTIR) confirmed the negligible role of π–π interactions in adsorption. This study offers fundamental insights into the mechanistic aspects of MB adsorption on lignin, laying the groundwork for potential modifications to enhance the performance of lignin-based adsorbents. The findings underscore the importance of hydroxyl groups and provide a roadmap for future studies examining the influence of steric factors and interactions with other organic molecules.

## 1 Introduction

Lignin is the second most abundant biopolymer on Earth. It is a complex network consisting of phenylpropane units, namely, p-hydroxyphenyl (H), guaiacyl (G), and syringyl (S). These are bonded through C–C (e.g., β–β, β-5, and β-4, 5–5′) and C–O interunit (e.g., β-O-4, 4-O-5, and α-O-4) linkages.^1,2^ Due to its structural features, lignin is rich in different functionalities: aliphatic (including benzylic) and phenolic hydroxyls, carbonyl, and carboxyl groups, and aromatic rings. The relative content of the different subunits and functional groups depends on the lignin source of origin, the conditions it has been exposed to, and the process by which it is extracted from biomass; therefore, there is a great variety of lignins. These include lignosulfonates, soda lignin, organosolv lignin, hydrolyzed lignin, and, the most prevalent, kraft lignin, among others. Such diversity gives lignin an enormous potential to replace a significant range of organic-based materials currently obtained from fossil (nonrenewable) sources.^3^

However, the structural complexity of lignin is one of the main challenges for its valorization into advanced materials, even simple ones such as adsorbents.^1^ Despite its rich oxygen content, unmodified lignin shows poor adsorption of metal ions^4^ due to inter- and intramolecular H-bonding in the macromolecule reducing its ability to interact with metal ions.^5^ However, lignin has been extensively recognized for its efficacy in removing toxic organic molecules, notably synthetic dyes.^6−11^ Unmodified lignin exhibits relatively high adsorption capacities for nitrogen-containing dyes, such as Methylene Blue (MB),^6−8^ Brilliant Red HE-3B^9^, reactive blue 21,^10^ and 2,4-dinitroanisole.^11^ The modification of lignin impacts the adsorption of dyes, attributed to alterations in lignin’s H-bonding capacity and the introduction of new adsorption sites.^12^ Common modifications of lignin encompass its deposition onto inorganic substrates such as Si^13^ and Fe^14,15^ oxides, chemical alterations via the grafting of functional groups containing oxygen, nitrogen, or sulfur,^12^ and pretreatments involving phenolation, demethylation, hydroxymethylation, reduction, or oxidation.^16^

The efficiency of the adsorbent in removing contaminant species relies on interactions between the adsorbate and the functional groups of the adsorbent. There are various types of these interactions, but generally, they can be described as the affinity between electron-deficient and electron-rich functional groups.^5^ The adsorption mechanism directly mirrors the nature of these intricate interactions. Understanding the mechanism of adsorption enables the enhancement of the adsorbent efficiency by regulating the number of active groups present in the material.

There have been several attempts to ascertain the mechanism of binding of various molecules to lignin.^17,18^ However, in much of the literature, the mechanism is either not thoroughly studied or speculated upon based on general knowledge or conjectures. This has led to the unsubstantiated acceptance that the adsorption of organic molecules to lignin (in aqueous solutions) occurs through lignin’s functional groups (−OH, −CO, and −COOH), H-bonding, and π–π interactions.^18^ However, as of the current time, there has not been a comprehensive investigation to establish the adsorption mechanism of organic molecules on lignin. In our recent study,^19^ we proposed an approach to elucidate the correlation between lignin structure, properties, and performance by selectively masking one specific lignin functionality at a time. This approach allowed for quantitative estimation of the adsorption activity of each functional lignin group. Herein, we used the approach to qualitatively and quantitatively estimate the interaction mechanism between MB and technical kraft lignin (Indulin AT).

## 2 Experimental Section

### 2.1 Chemicals and Reagents

Methylene Blue (MB), sodium hydroxide (NaOH, 99%), and hydrochloric acid (HCl, 37%) were purchased from Sigma-Aldrich. Deionized water was used to prepare the aqueous solutions. The softwood kraft lignin (Indulin AT, assigned as *Ind*) and its modified derivatives were used in the current work.

### 2.2 Synthesis of the Modified Ind lignin’s

**Figure 1:**
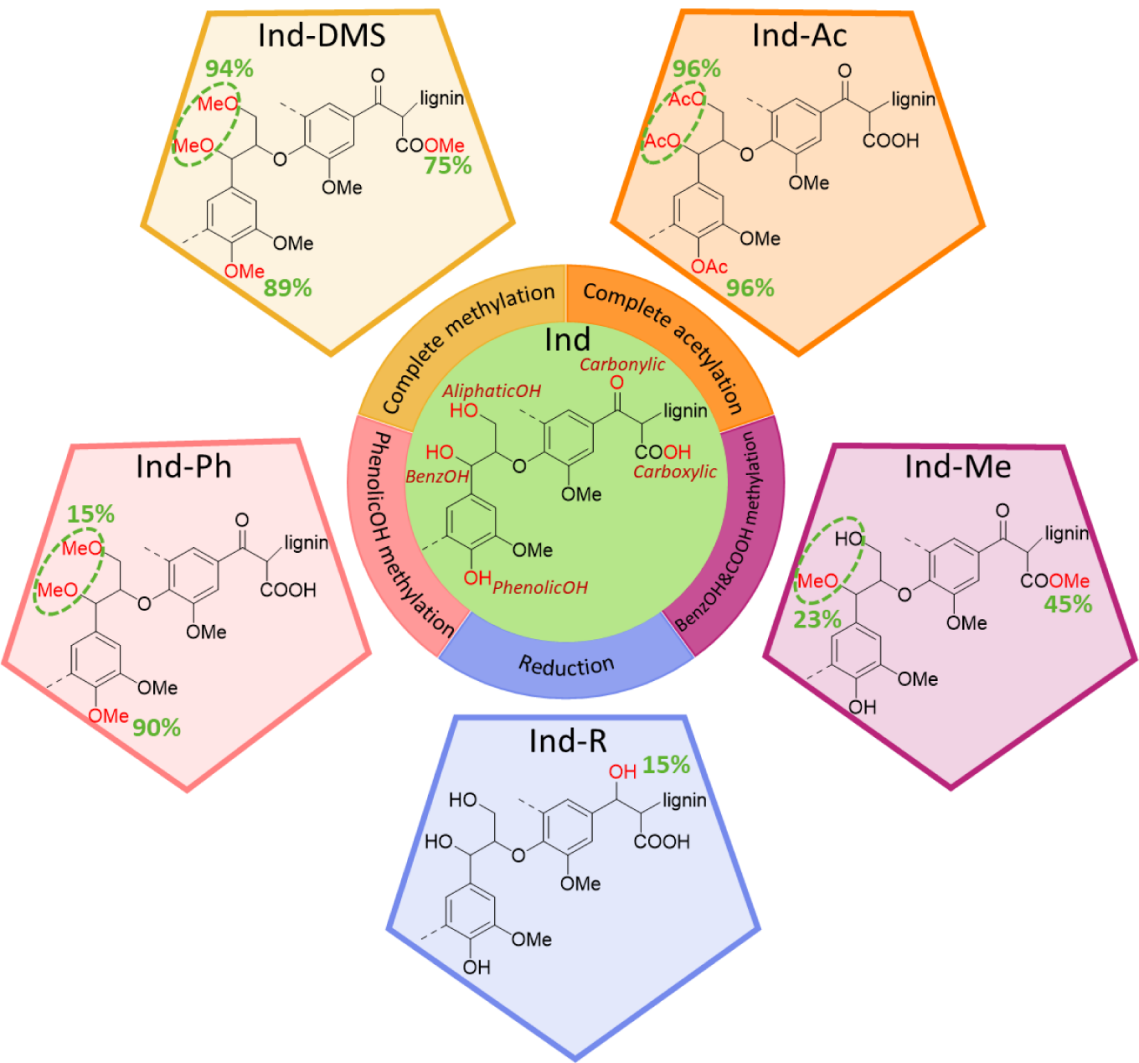
Schematic representation of Indulin-AT and its derivatives with blocked functional groups. The conversion efficiency is given in green next to the corresponding structures.

Detailed information about the preparation procedures and characterization of the modified lignin samples was presented in our previous work.^19^ Here, the most important details are briefly given in Figure 1.

Complete methylation of all −OH/–COOH groups of lignin, sample “*Ind-DMS,*” was performed by the reaction between *Ind* and dimethyl sulfate (DMS) in a highly concentrated (>30%) NaOH solution, according to the procedure proposed by Zakis.^20^ The acetylation of *Ind* was carried out in pyridin to mask all hydroxyl groups (sample *Ind-Ac*).^20^ Selective methylation of the benzylic hydroxyl groups of *Ind* and the partial esterification of carboxylic groups (sample *Ind-Me*) was achieved by reacting with acidic methanol in an anhydrous dioxane solution.^20^ To specifically methylate the phenolic −OH groups (sample *Ind-Ph*), *Ind* and DMS were heated at 80 °C in 0.7 M NaOH, pH 11–11.5.^21^ The reduction of lignin carbonyl groups (sample *Ind-R*) was conducted by reacting with NaBH_4_ in a basified ethanol–water mixture according to the method proposed by Zakis.^20^

These modifications of *Ind* produced a set of lignin samples with different functional group contents (shown in Figure 1). Overall, the set of lignin samples with similar structural characteristics but different available functional groups were synthesized, laying the foundation for the mechanistic study of organic dye adsorption.

### 2.3 Investigating the Adsorption Performance of Lignin

To evaluate the adsorption performance of lignin, the modified and original *Ind* samples were allowed to adsorb to MB and then subjected to spectroscopic analyses.

#### 2.3.1 Batch Adsorption Study

The pH of the points of zero charge (pH_pzc_) for *Ind* and *Ind-R* (the materials with the highest adsorption capacities according to our previous study)^19^ was evaluated by the pH drift method. 0.01 g of each lignin sample was added to 5 mL of 0.01 mol L^–1^ NaCl with an initial pH (pH_i_) controlled by adding 0.01 mol L^–1^ HCl or 0.01 mol L^–1^ NaOH to the solution. The system was shaken for 24 h, and the final pH (pH_e_) of the solution was measured.

*Methylene Blue Adsorption experiments* were carried out to evaluate unmodified and modified lignins’ adsorption performance using the batch technique. Precisely 0.05 g of each lignin sample was suspended in a solution of MB with a known initial concentration (adsorbent dosage 2 g L^–1^). The system was then shaken for 20 h (for kinetics study see below) in an Orbital Shaker INC/REFRIG 5000IR at 25 °C. Then, the liquid and solid phases were separated by centrifuging at 3374 × g for 10 min, and the residual MB concentration was determined in a UV-3100PC spectrophotometer at 664 nm. The removal efficiency (*R*, %) and specific concentration of the adsorbed MB (*q*_*e*_, mol g^–1^) were calculated according to eqs 1 and 2:
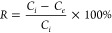
1
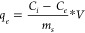
2where *C*_*i*_ and *C*_*e*_ are the initial and equilibrium MB concentrations,*m*_*s*_ (g) is the weight of the lignin sample, and *V* (L) is the volume of the initial dye solution. Each test was replicated three times.

*Kinetics experiments* were performed with *Ind* and *Ind-R*, as both lignin samples have the highest content of surface −OH/–COOH groups (according to the material characterization results presented in Figure S1) and demonstrated higher adsorption abilities in our previous work.^19^ Suspensions with known initial concentrations of MB cations (44.4 mg L^–1^ and 88.8 mg L^–1^ for *Ind* and 88.8 mg L^–1^ for *Ind-R*) and 2 g L^–1^ of lignin were shaken, and the equilibrated MB concentrations were determined between 10 min −24h.

The adsorption capacity of *Ind* and *Ind-R* materials was evaluated by an isotherm study. The experiments were carried out with varying concentrations of MB up to 160 mg L^–1^. Other lignin samples were tested at 8.88 mg L^–1^ and 44.4 mg L^–1^ of MB.

#### 2.3.2 Adsorption Kinetics and Equilibrium Models

The kinetic data of MB adsorption were fitted by using four models (pseudo-first-order (PFO), pseudo-second-order (PSO), mixed 1,2-order eq (MOE), and intraparticle diffusion). The PFO and PSO were used in nonlinear and linear forms. The equations of the applied models are as follows:

the pseudo-first-order:
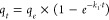
3the pseudo-first-order linear form:
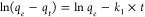
4the pseudo-second-order:
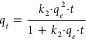
5the pseudo-second-order linear form:
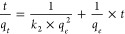
6the mixed 1,2-order:
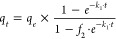
7

Weber and Morris equation:
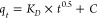
8where *q*_*t*_ and *q*_*e*_ (mg g^–1^) are the specific concentrations of MB cations adsorbed at time *t* (min) and equilibrium, *k*_*1*_ (min^–1^) and *k*_*2*_ (g mg^–1^ min^–1^) are PFO and PSO rate constants, *K*_*D*_ is the intraparticle diffusion rate (mg g^–1^ min^–0.5^), and *C* is a constant (mg g^–1^).

The experimental equilibrium data were fitted by applying Langmuir (LM, eq 9), Freundlich (FM, eq 10), and Langmuir–Freundlich (LFM, eq 11) models:
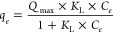
9
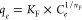
10
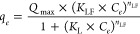
11where, *Q*_max_ (mg g^–1^) is the maximum adsorption capacity of the lignin sample, *K*_L_ (L mg^–1^), *K*_F_ (L^1/*n*F^ mg^1–1/*n*F^ g^–1^), and *K*_LF_ (L mg^–1^) are Langmuir, Freundlich, and Langmuir–Freundlich equilibrium constants, *n*_F_ and *n*_LF_ are the dimensionless exponents of Freundlich and Langmuir–Freundlich models related to the heterogeneity of the adsorption process. Nonlinear fitting of equilibrium data was performed using Microcal Origin (2019) software.

The statistical adequacy of the models was estimated according to Lima et al.^22^ by using three parameters—the adjusted determination coefficient (), standard deviation of residues (*SD*), and Bayesian information criterion (BIC):
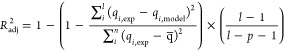
12
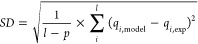
13
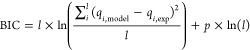
14where *l* is the total number of experimental points, *p* is the number of fitting parameters, *i* refers to the index of experimental/model parameters, *q*_i,model_ and *q*_i,exp_ are calculated and measured properties (*q*_*t*_ for kinetic and *q*_*e*_ for equilibrium models),  refers to the mean experimental value. The values  closer to 1.0 and the lowest SD are attributes of the best-fit model. If two models have very close values of parameters  and *SD*, criterium BIC is used to estimate whether the difference between the models is significant or whether both models provide fitting within the permissible deviations: if the the difference is <2, no significant difference between the models; if the difference lies between 2 and 6, then the model with lower BIC shows a positive perspective; if the BIC values differ within 6–10, then it is a strong possibility the model with a lower BIC value would be the best one; if the variations of BIC values are higher than 10, then the model with the lower BIC value provides better fitting.

#### 2.3.3 FTIR and X-ray Photoelectron Spectroscopy

Spectroscopic methods were used to investigate the lignin samples before and after the adsorption of MB to elucidate the possible mechanism of the interactions. FTIR spectra were recorded in ATR mode on a Bruker Tensor 27 ATR-FTIR Spectrometer (Bruker). The FTIR curves were normalized to peak at 1030 cm^–1^, attributed to aromatic C–H in-plane deformation (G > S), C–O deformation in primary alcohols or its ether form, and C=O stretch (unconjugated)^23^ since these groups do not contribute to the adsorption process. X-ray photoelectron spectroscopy (XPS) was performed using a PHI Quantera II Scanning XPS Microprobe (Physical Electronics). The high-resolution spectra were collected with an X-ray source of monochromatized Al Kα operated at 25.4 W and the passing energy of the hemispheric analyzer at 50 eV. An ion gun performed the surface charge compensation. The results were analyzed with MultiPak software (Physical Electronics). The spectral binding energy scale was calibrated taking the C 1s peak at 284.8 eV as a reference. Gauss–Lorentz peak profiles (90% Gauss) were used for spectral deconvolution.

## 3 Results and Discussion

### 3.1 Adsorption Ability of the Modified Lignin Samples

**Figure 2:**
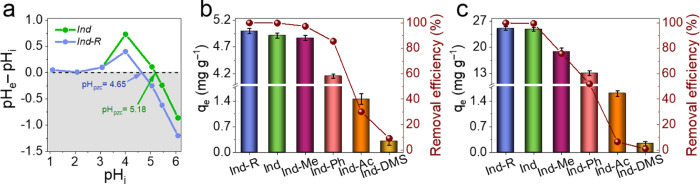
Comparison of (a) pH_pzc_ of *Ind* and *Ind-R*; (b) and (c) the adsorption behavior of bare and modified Ind in the solution containing 8.88 mg L^–1^ and 44.4 mg L^–1^ of MB, respectively.

The reactivity of the adsorbent significantly depends on the functional groups that it possesses. In the case of lignin, it is determined by the presence of hydroxyl (aliphatic (AlipOH) including benzylic (BenzOH) and phenolic (PhOH)) and carboxylic groups. These groups are anionic, so the lignin surface is negatively charged at high pHs due to the dissociation process; while at low pHs, protonation via H-bonding with water molecules occurs, resulting in a positively charged surface. The pH drift method and pH_pzc_ are typically used to estimate the point of zero charge, PZC (the pH of the solution at which the net charge on the lignin surface is zero). The pH_pzc_ was evaluated for *Ind* and *Ind-R* since both have the highest number of functional groups. The net surface charge becomes negative when the pH is above 5.18 for *Ind* and 4.65 for *Ind-R*. In Figure 2a, the *Ind-R* curve was shifted to the left compared to the *Ind* sample, which indicates the higher acidity of the *Ind-R* surface. The reduction process of carbonyl groups to create aliphatic −OH and increase phenolic −OH groups (the total −OH group content was found to be equal to 6.68 mmol g^–1^ in *Ind-R* compared to 6.02 mmol g^–1^ in *Ind*) resulted in a greater negative charge on the surface of the *Ind-R* sample. Further analysis showed that both lignin samples increased the pH of the solutions to values close to the pH_pzc_ retaining a minor negative charge on the surface. Thus, the lignin samples show a buffering capacity similar to organic acids and their anions. This trend was observed in all of the lignin derivatives tested in this study. Therefore, in adsorption experiments, the lignin surface not only provides adsorption centers but also buffers the solution close to the pH_pzc_. The negatively charged surface could enhance the adsorption of MB cations if electrostatic interactions are a major force for MB capture, promoting the dissociation of surface functional groups. Due to the performance of the lignin surface as a “buffer,” the surface charging effect can be thought of as negligible. This statement is correct only if no additional buffers are present within the system. In this work, the adsorption of MB on *Ind* and *Ind-*derivatives was performed from solutions that only contain MB, allowing the functional groups present in each derivative to buffer the pH at a level corresponding slightly negative charge on the lignin surface. Thus, this ensured the similarity of the lignin sample surfaces.

Investigations of the adsorption performance of the lignin samples with masked functional groups showed that there is a difference in their ability to remove the dye. From Figure 2b, the initial *Ind* could adsorb 99.86% of MB^+^ with an initial concentration of 8.88 mg L^–1^. The removal efficiency was slightly decreased for *Ind-Me* (97.31%) and drastically decreased for *Ind-Ph* (85.51%) and even further for *Ind-Ac* (30.04%) and *Ind-DMS* (9.19%). *Ind-R* had an improved adsorption performance with a removal efficiency of 99.98%.

The same trend was observed when the MB^+^ concentration was increased to 44.4 mg L^–1^ (Figure 2c). The near complete blocking of all −OH groups, by both methylation and acetylation, resulted in a more than 97% decrease in the adsorption activity of the samples, under the conditions tested. This indicates that hydroxyl groups are crucial for lignin sorption activity and the negligible role of other functional groups (−COOH and −CO). The differences between completely methylated *Ind-DMS* and the partially methylated *Ind-Me* and *Ind-Ph* samples show that there could be about 50:50 split in the sorption to phenolic and aliphatic hydroxyls. Since the role of −COOH groups was assumed to be negligible, an ∼25% decrease in the adsorption of MB by *Ind-Me* was attributed predominantly to the conversion of AlipOH groups (particularly BenzOH) into methoxy. Overall, the contribution and the activity of each functional group to the adsorption of MB could be represented by the following trend: BenzOH> −COOH > AlipOH > PhOH, which was presented and discussed in our previous work.^19^ Interestingly, there is practically no adsorption of MB on *Ind-DMS*, where there is almost complete blocking of all of the functional groups. This suggests that π–π interactions play almost no role in MB adsorption and confirms that the functional groups of the lignin play the valuable role in the physicochemical interactions of lignin surface with MB.

### 3.2 Kinetics and Equilibrium Study of MB Adsorption

Since the hydroxyl groups of lignin are crucial in determining their sorption activity, the samples with the highest −OH group contents, *Ind-R* and *Ind*, were chosen to investigate the kinetic and equilibrium parameters of the adsorption.

#### 3.2.1 Kinetics Study

Kinetics studies were performed to determine the adsorption rate and dynamic behavior of the system. In general, the overall rate of the adsorption process is determined by the slowest of the three processes involved in the adsorption of molecules from the aqueous phase onto a solid: film diffusion, intraparticle diffusion, and adsorption onto the surface of the adsorbent. Film diffusion involves the transport of the adsorbate from the bulk phase to the exterior surface of the adsorbent, whereas intraparticle diffusion refers to transport into the adsorbent through pores or surface diffusion.

**Figure 3:**
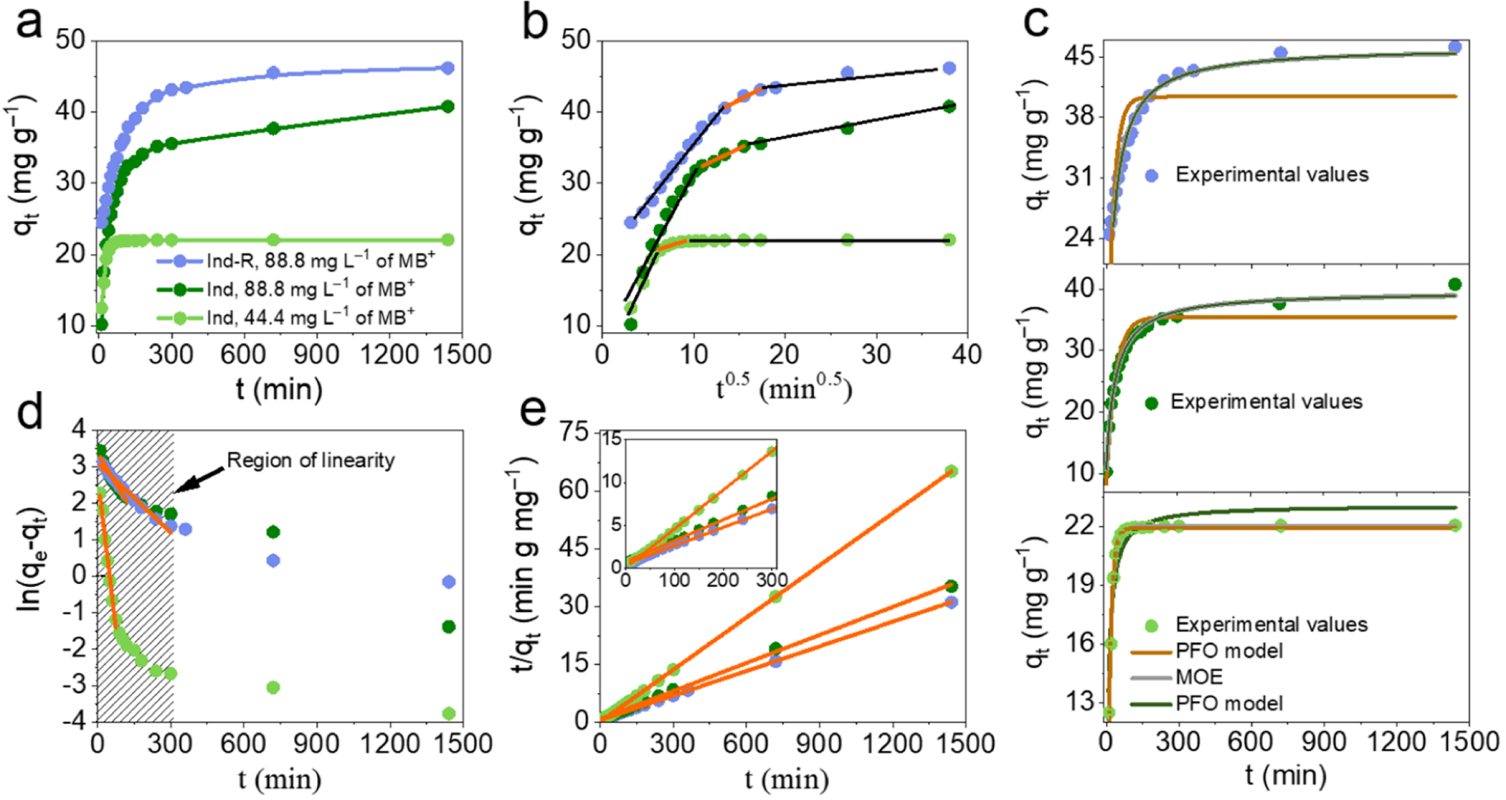
(a) Influence of contact time on MB dye adsorption on *Ind* and *Ind-R*; (b) intraparticle diffusion model of MB adsorption; (c) nonlinear fitting of MB adsorption kinetics; (d) pseudo-first-order linear plots; (e) pseudo-second-order linear plots.

Determination of the adsorption mechanism and the adsorption rate is required to assess the suitability of the sorbents for large-scale applications. Adsorption tests to determine the kinetic parameters began with an analysis of how phase contact time influences the process. The results of these experiments are listed in Figure 3a.

In all cases presented in Figure 3a, the adsorption shows two distinct phases. The initial faster stage takes place in the first hour in low (44.4 mg L^–1^) MB concentrations and 3 h at higher MB concentrations (88.8 mg L^–1^). In the *Ind* sample, 50% of MB was removed in the first 35 min, and equilibrium was reached after 24 h. For the *Ind-R*, 50% of the MB was removed after 9 min and the equilibrium was obtained after 12 h. It can therefore be concluded that MB has a higher affinity for the surface of the *Ind-R* sample.

Several kinetic models were used to fit these data and determine the rate-controlling step and the rate of the adsorption process. A plot of *q*_*t*_*vs t*^0.5^ was used to understand the impact of the intraparticle diffusion mechanism on the MB adsorption, Figure 3b. The curves have three linear regions. The first linear region corresponds to the fast adsorption process, which involves the transport of MB across the near-surface layer of lignin, followed by the bonding of MB on the external surface of lignin. The second linear region (marked by the orange line in Figure 3b) corresponds to the intraparticle diffusion process, the transport of MB species to the pores of lignin, and can be described by the Weber–Morris intraparticle diffusion model eq 8. The calculated values of K_D_ were 0.61 ± 0.05 mg g^–1^ min^–^°.^5^ and 0.66 ± 0.09 mg g^–1^ min^–^°.^5^ for *Ind* and *Ind-R*, respectively. The resemblance in the parameters of the Weber–Morris models for both materials suggests a close resemblance in the intraparticle diffusion process. This similarity can be attributed to the low porosity of lignin, which also explains the minor contribution of the last kinetic stage (the third linear region)—the diffusion of adsorbate species into micropores of the adsorbent. Therefore, the results described above demonstrate that the adsorption rate is affected more by interactions between MB and the surface of lignin than by diffusion of MB.

The PFO and PSO kinetic model parameters were obtained by fitting them to the nonlinear (Figure 3c) and linear (Figure 3d,e) models, and the fitted parameters are listed in Table 1. Lower SDs and higher  values were observed for nonlinear fitting. Lima et al.^24^ have reported that the linearization of kinetics (equilibrium) data can lead to overestimating the fitted values. This is mainly due to postulating the same variance of all of the Y values in the linear fit. However, the values of *q*_t_ or *q*_e_ usually have different variances. Considering all this, only nonlinear fitting will be further applied in this study. Kinetic studies in low MB concentration solutions display first-order kinetics. This is attributed to the fact that this MB concentration lies in the linear region of the isotherm—Henry’s range.^25^ Adsorption kinetics at 88.8 mg L^–1^ of MB^+^ (the point near the adsorption equilibrium range) displayed second-order kinetic behavior for both lignin materials tested. The SDs and  when fitting the low MB concentration data to PFO and PSO models seem to suggest that there is no significant difference between the two or that parallel first- and second-order processes are occurring (*SD* changed from 0.386 to 0.766 and  from 0.979 to 0.919 for PFO and PSO, correspondingly). However, despite the small variations in values of these two criteria, the difference in BIC parameters (−27.1 vs −5.1) indicates that the PFO still fits better experimental data than PSO. For strong confirmation of the hypothesis, the MOE model was applied (Figure 3c). In the fitted parameters, Table 1, *f*_*2*_ is close to 1 (0.995 for *Ind* and 0.992 for *Ind-R*), indicating that the second-order process is dominating at higher concentrations. It is an agreement to the fitting results with PFO and PSO that the latter is the best model at the higher MB concentration. At the lower MB concentration, *f*_*2*_ was 0.45, indicating a mixed kinetic process where the first-order process dominates.

**Table 1:** Fitted Kinetic Model Parameters for the Adsorption of MB on *Ind* and *Ind-R*

Kinetic model	Parameter symbol, unit	*Ind*		*Ind-R*
		concentration of MB^+^, mg L^–1^		
		44.4	88.8	88.8
	*q*_*e*,exp_, mg g^–1^	22.2 ± 0.2	40.9 ± 0.3	46.5 ± 0.3
pseudo-first-order model (linear fitting)	*q*_*e*,cal_, mg g^–1^	16 ± 1	29 ± 2	21.8 ± 0.1
	*k*_1_, min^–1^	0.055 ± 0.002	0.011 ± 0.001	0.006 ± 1
	*	0.984	0.954	0.977
	*SD*, mg g^–1^	6.5	11.9	26.7
pseudo-second-order model (linear fitting)	*q*_*e*,cal_, mg g^–1^	22.2 ± 0.2	41.2 ± 0.2	46.7 ± 0.2
	*k*_2_, g mg^–1^ min^–1^	0.017 ± 0.001	7.1 × 10^–4^ ± 3 × 10^–5^	8.5 × 10^–4^ ± 2 × 10^–5^
		0.9999	0.9991	0.9997
	*SD*, mg g^–1^	1.69	1.27	1.65
pseudo-first-order model (nonlinear fitting)	*q*_*e*,cal_, mg g^–1^	21.9 ± 0.1	35.5 ± 0.8	40 ± 1
	*k*_1_, min^–1^	0.070 ± 0.002	0.026 ± 0.002	0.039 ± 0.007
		0.979	0.926	0.553
	*SD*, mg g^–1^	0.386	2.153	4.615
	BIC	–27.1	28.0	55.5
pseudo-second-order model (nonlinear fitting)	*q*_*e*,cal_, mg g^–1^	23.1 ± 0.3	39.6 ± 0.4	46.1 ± 0.5
	*k*_2_, g mg^–1^ min^–1^	6.1 × 10^–3^± 4 × 10^–4^	9.3 × 10^–4^ ± 4 × 10^–5^	8.8 × 10^–4^ ± 4 × 10^–5^
		0.919	0.993	0.974
	*SD*, mg g^–1^	0.766	0.663	0.951
	BIC	–5.1	–9.7	1.8
mixed 1,2-order rate equation model (nonlinear fitting)	*q*_*e*,cal_, mg g^–1^	22.0 ± 0.1	39.5 ± 0.4	46.0 ± 0.8
	*k*_1_, min^–1^	0.051 ± 0.006	1.66 × 10^–4^ ± 1 × 10^–5^	3.2 × 10^–4^ ± 9 × 10^–5^
	*f*_*2*_	0.45 ± 0.09	0.995 ± 0.001	0.992 ± 0.003
		0.989	0.992	0.972
	*SD*, mg g^–1^	0.279	0.689	0.991
	BIC	–35.8	–6.9	4.5
intraparticle diffusion model	*K*_*D*_, mg g^–1^·min^–^°.^5^	0.34 ± 0.9	0.61 ± 0.05	0.66 ± 0.09
	*C*, mg g^1^	18.8 ± 0.6	25.7 ± 0.6	32 ± 1
		0.896	0.983	0.948

The fitted parameters for 44.4 mg L^–1^ MB fit to a PFO model and 88 mg L^–1^ MB fit to PSO were used to estimate the initial sorption rate *h*_0_ (mg g^–1^ min^–1^) according to the equation proposed by Ho:^26^
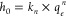
15where *k*_*n*_ is the rate constant [min^–1^ (g mg^–1^)^n–1^], *q*_*e*_ is the adsorbed MB at equilibrium (mg g^–1^), and *n* is the order of the kinetic model. For *Ind*, the calculated values were 1.53 ± 0.05 mg g^–1^ min^–1^ for the low concentration and 1.46 ± 0.09 mg g^–1^ min^–1^ for the high concentration, while for *Ind-R* the rate was found to be 1.87 ± 0.02 mg g^–1^ min^–1^ (only the high concentration tested). Comparing the average values of the rate for *Ind* (1.50 mg g^–1^ min^–1^) with the one for *Ind-R*, this equates to a 20% rate increase. This correlates to the increase in the hydroxyl group content during the reduction of *Ind* (+13.3% AlipOH and +8.4% of PhOH). These results further confirm the importance of hydroxyl groups in bonding the MB and highlight their key role in the kinetics of the adsorption process.

Excellent correlation to the PSOE, mixed 1,2-order rate equation, and the nonlinear dependency of the IPD model indicated that the adsorption process is limited by the adsorption forces due to specific interactions to a higher extent, rather than by MB diffusion and mass transfer of the MB to the adsorption sites of *Ind* and *Ind-R*. Moreover, the adsorption rate is dependent on the adsorption capacity of the selected materials rather than on the concentration of MB dye. However, at lower MB concentrations (44.4 mg L^–1^), the process becomes mass transport limited, as confirmed by good agreement with the PFOE.

#### 3.2.2 Equilibrium Study

**Figure 4:**
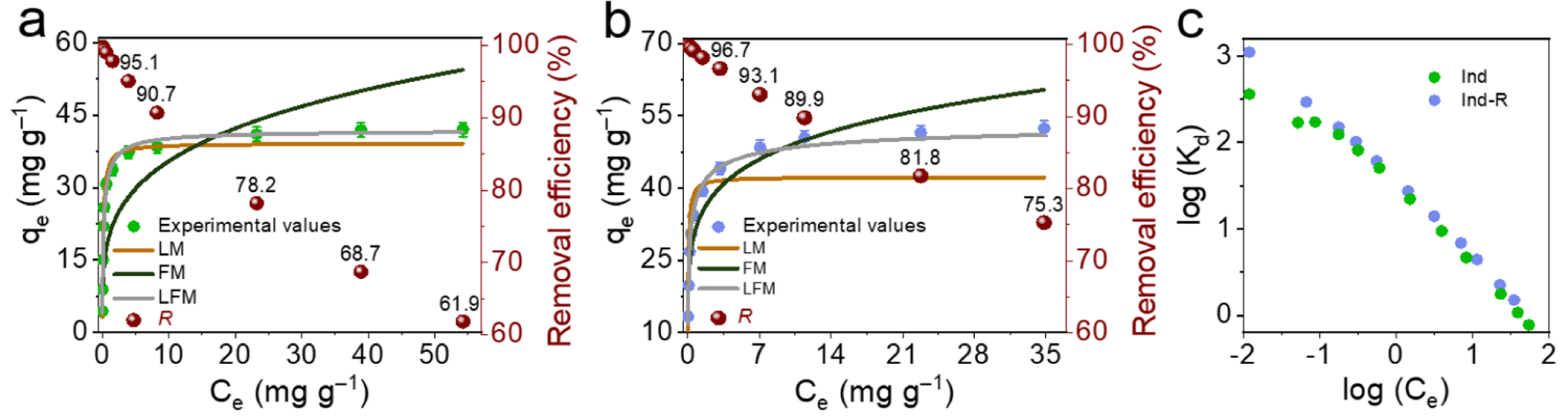
Adsorption isotherms of MB onto (a) *Ind* and (b) *Ind-R*; (c) the change of the distribution constant log*K*_d_ of MB vs equilibrium concentrations of MB in the aqueous phase.

Adsorption isotherms of MB were used to study its adsorption equilibrium (Figure 4a for *Ind* and Figure 4b for *Ind-R*). From these data, *Ind-R* shows a 21% higher adsorption capacity than does *Ind*. The experimental removal efficiency reached 62% for *Ind* (Figure 4a) and 75% for *Ind-R* (Figure 4b) in solutions with the same initial concentration of MB, confirming their potential for MB adsorption.

The Langmuir, Freundlich, and Langmuir–Freundlich isotherm models were used to analyze the data. The characteristic parameters for each isotherm were determined and are summarized in Table 2. The experimental data for *Ind* and *Ind-R* fit the Langmuir–Freundlich isotherm model best, in they had the lowest SDs (1.93 and 2.69), highest correlation coefficients (*R*^2^: 0.9885 and 0.988) and corresponding BIC values differ more than 10 units vs others models. Fits of the experimental data with calculated adsorption capacity are shown in Figure 4a,b. The correlation of the experimental data to the Langmuir–Freundlich isotherm model confirms the complicated adsorption process that occurred on both the *Ind* and *Ind-R* surfaces. The Langmuir–Freundlich isotherm combines the simple monolayer adsorption model with the notable impact of surface heterogeneity. Both *Ind* and *Ind-R* can be thought of as heterogeneous adsorbents with energetically different sites distributed across their surfaces.^27^ This heterogeneity is common for systems containing organic compounds and highly interactive species. The maximum capacities were calculated as 42 and 55 mg g^–1^ for *Ind* and *Ind-R* samples, respectively. An increase in the adsorption capacity (+23.7%) and Langmuir–Freundlich constant (+19.7%) was observed when moving from *Ind* to *Ind-R.* This is attributed to the increasing number of functional groups, in agreement with the kinetic data. The Langmuir–Freundlich constant (*K*_LF_) of both materials is relatively high, indicating the complex nature of MB adsorption. Thus, it is most likely that several functional groups are participating in the adsorption process simultaneously. Moreover, the drastic linear drop of the log*K*_d_ constant with log c_eq_ (Figure 4c) confirms the strong interaction of the dye molecules with the surface of *Ind* and *Ind-R*. This also indicates a decrease in the number of strong sorption sites on the surface of the studied materials.

**Table 2:** Fitting Equilibrium Model Parameters for MB Adsorption on *Ind* and *Ind-R*

Sample	Parameter symbol					
	Langmuir model (eq 9)					
	*Q*_*max*_mg g^–1^	*K*_*L*_L mg^–1^			SD mg g^–1^	BIC
*Ind*	39 ± 2	7.1 ± 0.8		0.974	4.00	36.0
*Ind-R*	42 ± 4	19 ± 7		0.794	7.23	46.1
	Freundlich model (eq 10)					
		*K*_*F*_,L^1/nF^ mg^1–1/nF^ g^–1^	*n*_*F*_		SD mg g^–1^	BIC
*Ind*		20 ± 2	0.25 ± 0.01	0.785	11.5	61.4
*Ind-R*		33 ± 2	0.17 ± 0.01	0.941	3.87	32.4
	Langmuir–Freundlich model (eq 11)					
	*Q*_*max*_mg g^–1^	*K*_*LF*_L mg^–1^	*n*_*LF*_		SD mg g^–1^	BIC
*Ind*	42 ± 1	4.9 ± 0.5	0.80 ± 0.01	0.988	2.69	26.0
*Ind-R*	55 ± 2	6.1± 0.9	0.51± 0.09	0.985	1.93	17.1

### 3.3 Characterization of Lignin Samples with Adsorbed MB

**Figure 5:**
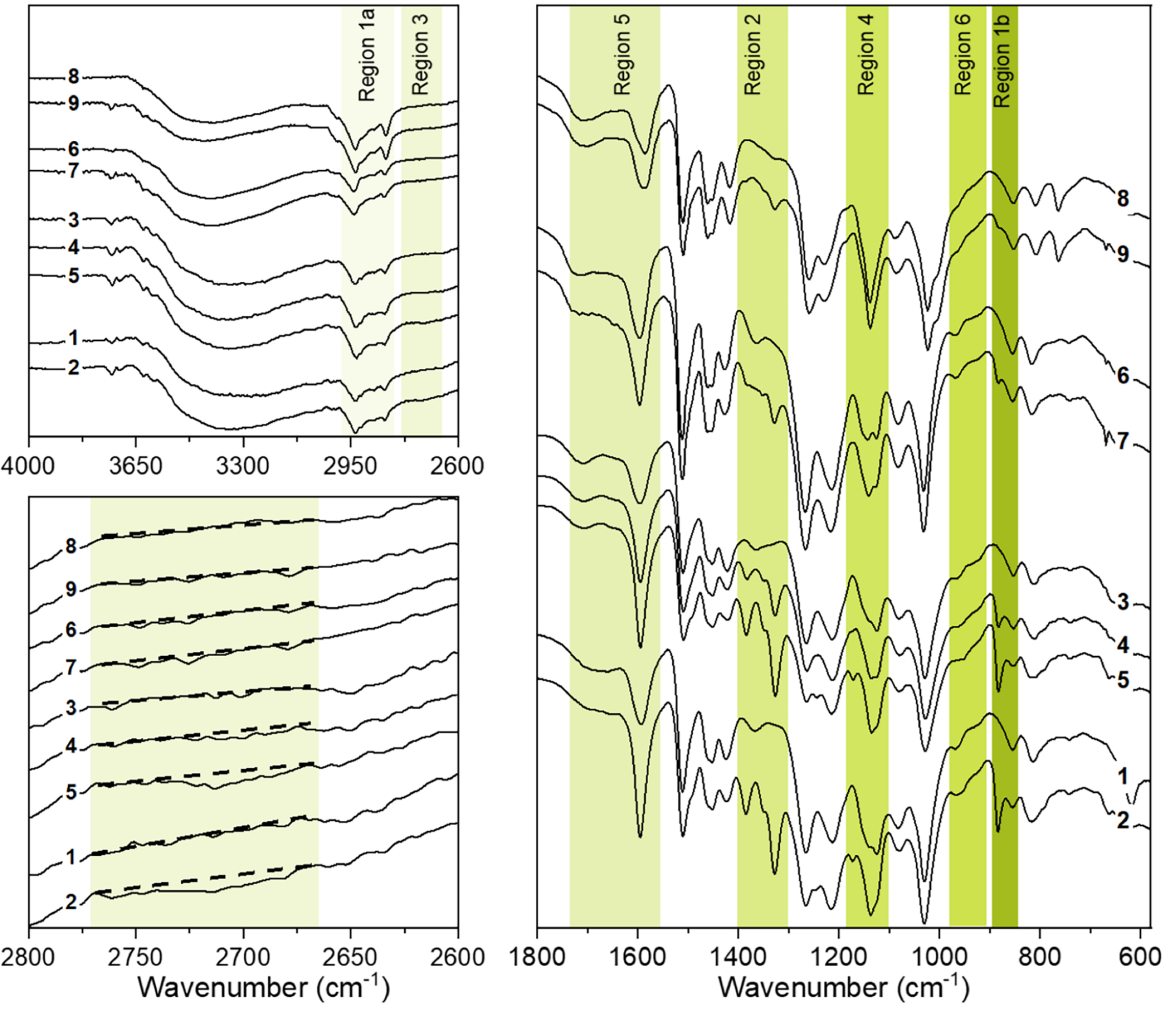
FTIR spectra *Ind* (1), *Ind-R* (3), *Ind-Me* (6) and *Ind-Ph* (8) and with MB adsorbed *Ind/MB-1* (2) (42.2 mg g^–1^ of MB), *Ind-R/MB-2* (4) (19.8 mg·g^–1^ of MB), *Ind-R/MB-3* (5) (55.1 mg g^–1^ of MB), *Ind-Me/MB-4* (7) (17.8 mg·g^–1^ of MB), and *Ind-Ph/MB-5* (9) (12.5 mg·g^–1^ of MB).

To confirm the findings from the adsorption study, FTIR spectra were recorded of *Ind*, *Ind-R*, *Ind-Me*, and *Ind-Ph* before and after the adsorption of MB (Figure 5). The adsorbed amounts of MB^+^ were 42.2 mg g^–1^ for *Ind/MB*-1, 19.8 mg g^–1^ for *Ind-R/MB*-2, 55.1 mg g^–1^ for *Ind-R/MB*-3, 17.8 mg g^–1^ for *Ind-Me/MB*-4, and 12.5 mg g^–1^ for *Ind-Ph*/*MB*-5. The FTIR spectra of lignin have been assigned and described by Faix et al.^23^ In this work, the only changes in the spectra affected by adsorption are listed in Table S1. The broad peaks between 3600 and 3000 cm^–1^, attributed to the hydroxyl groups stretching the −H, are present in the spectra of all the samples studied. The band intensity depends on the total concentration of −OH, which is highest for *Ind-R* and lowest for *Ind-Ph*, as expected. After the adsorption of MB, this peak became asymmetric and shifted to higher wavenumbers. This is due to the appearance of dissociated −O^–^ groups caused by the electrostatic interactions between the hydroxyl groups and MB cations. The coupled bands at ∼2935 cm^–1^ and ∼2837 cm^–1^ in region 1a (Figure 5) are assigned to asymmetric and symmetric C–H stretches in the methyl and methylene groups. Those in region 1b at ∼854 cm^–1^ and ∼812 cm^–1^ belong to C–H out-of-plane stretches in positions 2, 5, and 6 of G-units. After the adsorption of MB, the intensity of the bands in region 1a slightly increased due to the presence of the four methyl groups in MB. A new peak appeared at 883 cm^–1^ in region 1b and its intensity was dependent on the amount of MB in the sample. This band is assigned to C–H out-of-plane bending vibrations^28^ of the MB aromatic ring, and its satellite caused a small broadening of the peak at 814 cm^–1^. Region 2 of the spectra before adsorption has a band at ∼1365 cm^–1^, due to O–H bending of the phenolic fragments of lignin. This peak almost disappears in the spectrum of *Ind-Ph* since practically all phenolic hydroxyls were masked via the ether form with methoxy groups. When MB was adsorbed on the surface, the band shifted to 1385 cm^–1^ and its intensity increased. This is due to the formation of phenolate on the lignin surface caused by electrostatic interactions between the lignin and MB cations. The methyl group peaks of MB also increased in intensity due to C–H symmetric deformation in CH_3_.

Two additional peaks appeared in region 2 after MB adsorption. The presence of a small band at 1351 cm^–1^, ν_het_(C=S^+^),^29^ indicates this group did not participate in the bonding of MB to the lignin samples, as there is no change in its position compared with pure MB (Supporting Information). The second band was attributed to ν(C–N) stretching in N–CH_3_^30^ and grew with increasing MB loading. This band shifted from 1336 cm^–1^ in pure MB to 1327 cm^–1^ upon adsorption and could be the result of H-bonding between the −OH groups of lignin and the N atom of MB. To test this hypothesis, region 3 was investigated. The very weak band at 2713 cm^–1^, attributed to H-bonded ν(-N-(CH_3_)_2_,^30^ was observed for *Ind* and *Ind-R* lignins with the MB adsorbed. The absence of this band in the spectra of the other lignin derivatives loaded with MB could either be due to the lower concentration of MB adsorbed on these samples or masking of their aliphatic (including benzylic) groups.^19^

In region 4, before adsorption, a band at 1125–1135 cm^–1^ was observed and assigned to aromatic C–H in-plane deformation. After adsorption, the intensity of this band increased slightly due to additional C–Hs from the aromatic rings of MB. A new band at 1172 cm^–1^ is observed for lignin’s with a high amount of MB loading, this peak is characteristic of δ_het_(CH).^31^ Region 5 is rich in bands due to the presence of several functional groups contributing to the spectrum, which could lead to peak overlapping. A relatively strong band was observed at ∼1595 cm^–1^ in all of the lignin derivatives, attributed to aromatic skeletal vibrations and possibly to C=O stretching. This peak’s intensity increased after MB adsorption due to its overlapping with the ν_het_(C–N) and ν_het_(C–C) peaks. The broad band with a shoulder observed between 1765 and 1636 cm^–1^ is attributed to the C=O stretch. The differences in the position of this peak in different lignin samples are attributed to the different chemical environments of the carbonyls in each of the *Ind* derivatives. The shape of this band did not change after the adsorption of MB, and the deviations in its intensity were negligible (less than 6%). This suggests that the carbonyl groups play no role in MB bonding, and there is no formation of Shiff bases with the N atoms of MB. Region 6 in the lignin sample spectra before MB adsorption has two low intensity bands at ∼968 and 923 cm^–1^, assigned to —HC=CH— out-of-plane deformations and C–H out-of-plane (aromatic) stretches, correspondingly. However, a difference was observed after MB adsorption between lignin samples with free and masked −OH groups. For *Ind* and *Ind-R*, when MB was adsorbed, the peak at 923 cm^–1^ was smoothed. This smoothing was minor for *Ind/MB-1* and *Ind-R/MB-2* but complete for *Ind-R/MB-3*. This smoothing is a result of interactions between the N-hetero of MB and −OH of lignin, the superimposition of the N_het_•••HO (at 930–945 cm^–1^)^32^ band and the peak at 923 cm^–1^. The spectra of *Ind-Me/MB*-4 and *Ind-Ph*/*MB*-5 are almost identical, unlike those of *Ind-Me* and *Ind-Ph*. *Ind-R/MB-2* and *Ind-Me/MB*-4 have almost the same amount of MB adsorbed; however, *Ind-Me* has a smaller number of hydroxyl groups, due to masking of the benzylic −OH. Coupling this with the appearance of N_het_•••HO and -N-(CH_3_)_2_•••HO bands (region 3), indicate the aliphatic hydroxyls (including benzylic groups) H-bond to MB. The interactions between lignin carbonyls and aliphatic nitrogen are minor. To check the contributions of the π–π interactions, XPS was performed.

**Figure 6:**
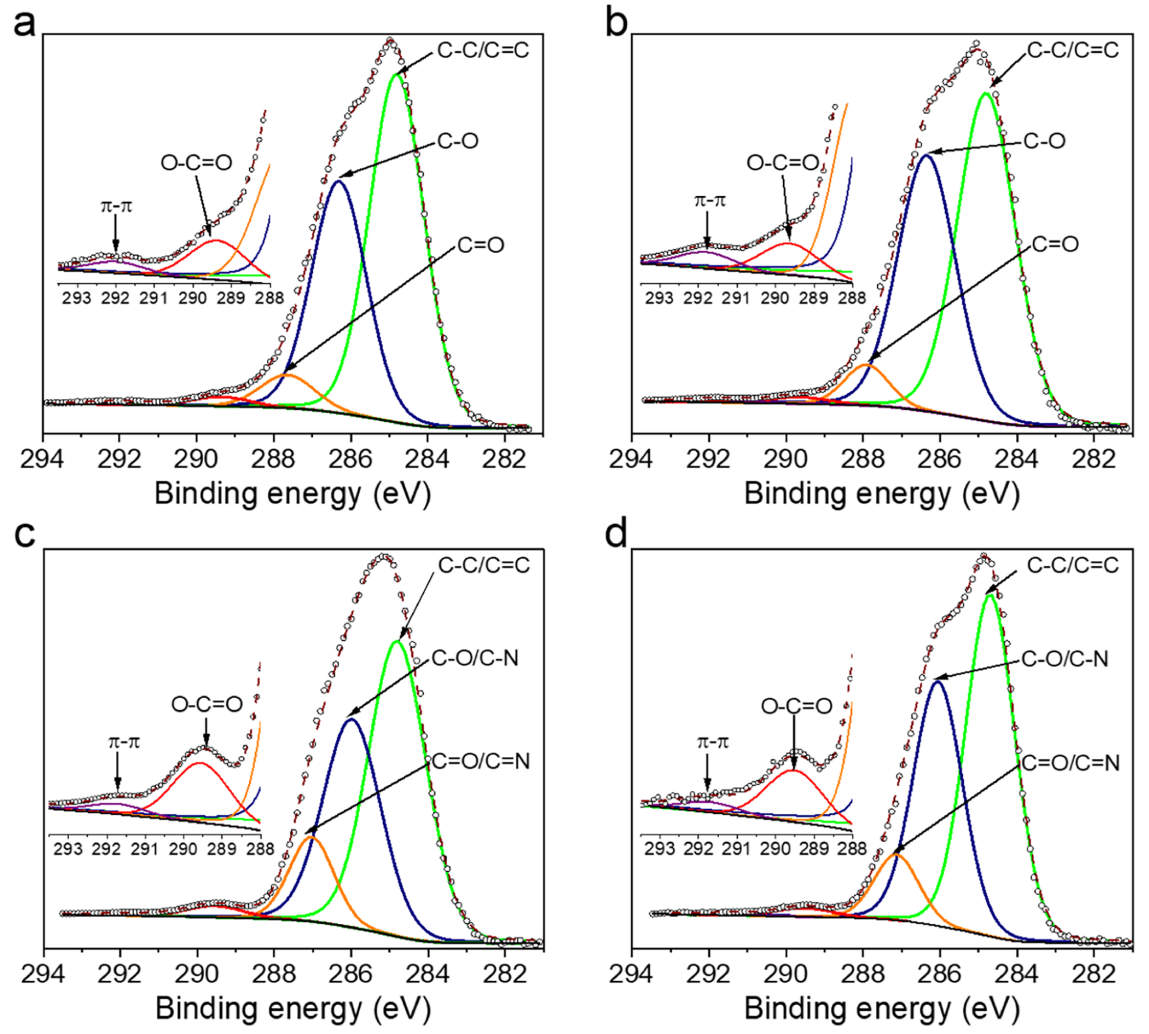
Deconvoluted XPS C 1s spectra: a) *Ind*, b) *Ind-R*, c) *Ind/MB-1* (42.2 mg g^–1^ of MB), and d) *Ind-R/MB-3* (55.1 mg g^–1^ of MB).

XPS spectra of *Ind* and *Ind-R* before and after the adsorption of MB (samples *Ind/MB-1* and *Ind-R/MB-3*) were recorded. The deconvoluted C 1s XPS spectra (Figure 6) contain five components: E_bind_ = 284.8 eV is assigned to C=C/C–C or C–H, E_bind_ = 286.0–286.4 eV characteristic of C–O and C–N, E_bind_ = 287.0–288.0 eV from C=O and C=N, and E_bind_ = 289.4–289.6 eV from the carboxyl carbon. The fifth peak from π–π interactions has a very low intensity (statistically not significant, area <1%) for both samples evidencing that there are no π–π interactions between MB and the lignin surface.

### 3.4 The Mechanism of MB Binding to the Lignin Surface

According to the data presented above, multiple interactions are present in the adsorption of MB on lignin. First, electrostatic and van der Waals interactions are present between the negatively charged groups of lignin, some through the −COOH but mainly through phenolic −OH groups, and MB^+^ cations. This is confirmed by FTIR spectroscopy, where phenolic −OH bands shift toward phenolates, and the O–H stretch in hydroxyl groups shifts toward higher wavenumbers after MB adsorption. Stronger interactions between the adsorbent and the adsorbate occur through H-bonding, specifically between the aliphatic and heterocyclic N atoms of MB and the aliphatic and benzylic hydroxyls of lignin. The participation of these hydroxyls in H-bonding was confirmed by the presence of the corresponding band in the FTIR spectrum *Ind-R/MB-2*, and its absence in *Ind-Me/MB-3,* where the benzylic hydroxyls were blocked. It was observed that π–π-interactions between MB and lignin are negligible in the adsorption process. This could be because of steric hindrances in the disordered macromolecular structure of the lignin.

To estimate the contribution of each interaction type in the total MB adsorption, several assumptions were made. Kubo and Kadla^33^ have shown that aliphatic hydroxyl (−OH) groups form stronger H-bonds than phenolic hydroxyl groups (PhOH). Since these hydroxyls act as H-bond acceptors and MB only has acceptor centers, it was assumed that phenolic hydroxyls only participate in the adsorption process via electrostatic interactions. Aliphatic hydroxyls (including benzylic hydroxyls) are weakly acidic, so they only act as H-bond donors. Carboxylic groups (−COOH) have an innate anionic nature and, despite their low concentration, could potentially capture cationic species. Considering all of this, MB adsorption on unmodified *Ind* lignin can be expressed as
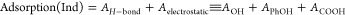
16where Adsorption(Ind) is the adsorption when all possible adsorption centers are fully occupied by the adsorbate; *A*_H-bond_ and *A*_electrostatic_ are the contributions of H-bonding and electrostatic attractions; *A*_OH_, *A*_PhOH_, and *A*_COOH_ are the total contributions of each of the respective functional groups. It should be noted that *A*_H-bond_ = *A*_OH_ and *A*_electrostatic_ = *A*_PhOH_ + *A*_COOH_.

**Figure 7:**
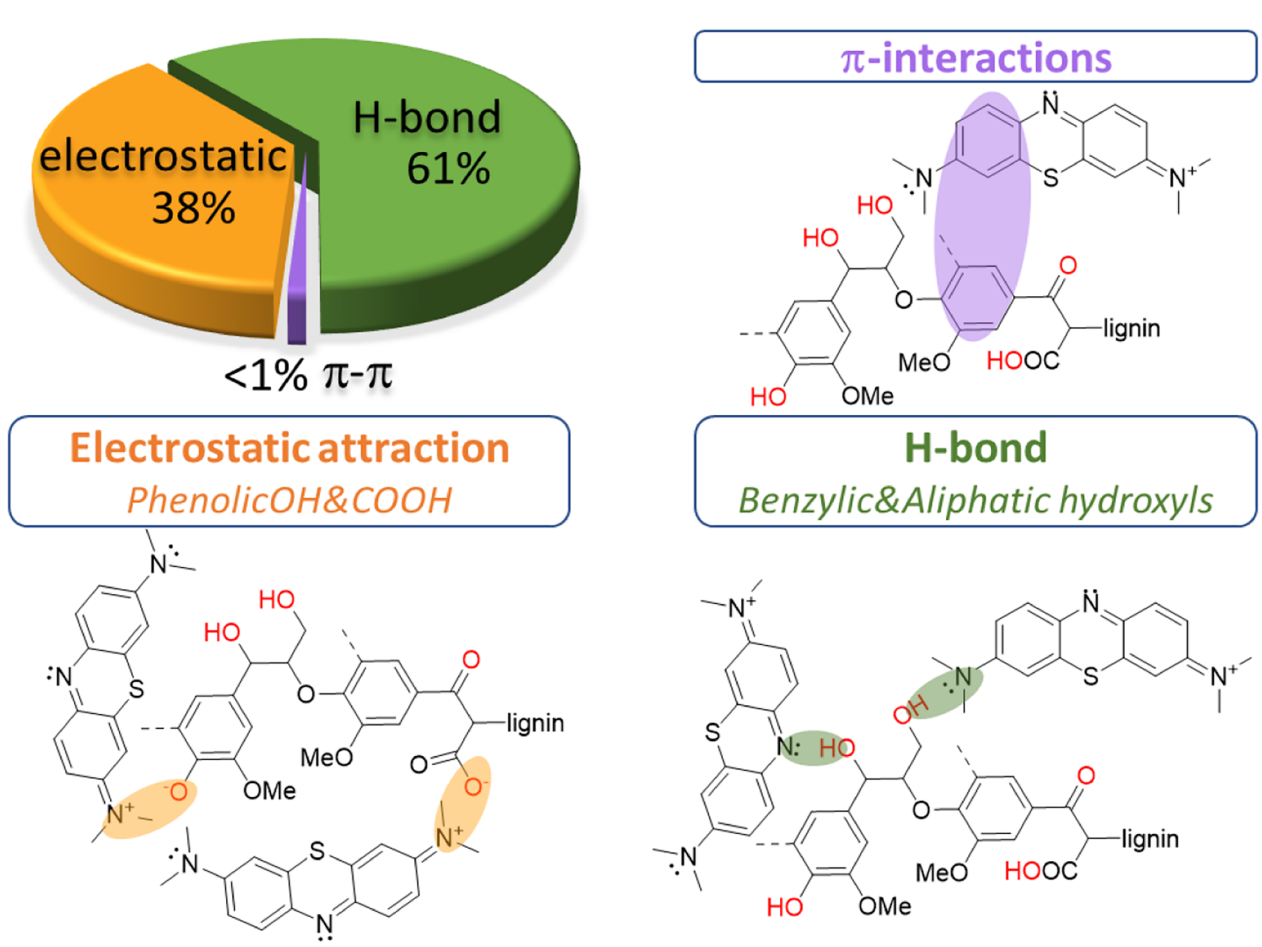
Proposed contributions of the different interactions between Indulin-AT and MB.

When all the adsorption centers are occupied, it could be difficult to separate the contribution of each mechanism, since other factors, such as cooperativity effects,^34^ can significantly influence adsorption. One solution to this problem is a “dilution” of the specific concentration of adsorption centers. When some functional groups are fully or partially blocked, a lignin surface with fewer active adsorption centers is obtained. This also increases the average distance between the adsorption centers and reduces cooperativity.^35^ Considering this, eq 16 can be modified for adsorption to such lignin samples:
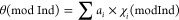
17where θ(modInd) is the ration of the extrapolated adsorption capacities of modified and initial lignin samples, *a*_*i*_ is the relative contribution efficiency ratio of each functional group type, *i* represents the functional group (−OH, PhOH or −COOH), and χ_i_(modInd) is the fraction of free functional groups (Figure 1) in the modified Indulin-AT. To estimate *a*_*i*_, eq 17 was solved for *Ind-Me* (θ = 0.44), *Ind-Ph* (θ = 0.30), and *Ind-Ac* (θ = 0.04). The values calculated were 0.32 for −OH, 0.19 for PhOH, and 0.012 for −COOH. From this, we determined that H-bonding accounts for 61% and electrostatic interactions 38% of adsorption. The adsorption mechanism of MB on lignin is summarized in Figure 7.

The discovered observations regarding the significant role of aliphatic OH in the interactions between lignin and Methylene Blue indicate the potential attraction of lignin-based composites with extremely high aliphatic hydroxyl content, such as lignin nanoparticles.^36^

## 4 Conclusions

In this work, the interactions between lignin and the cationic dye Methylene Blue were systematically investigated. This was done through the combination of an adsorption performance study of lignin samples and FTIR and XPS. The lignin samples with selectively blocked functional groups were used to estimate the functionality contribution to the total adsorption performance of unmodified lignin. Almost complete masking of all functional groups (residual content of aliphatic −OH, 6%; phenolic −OH, 11%; and −COOH, 25%) led to the near complete inability of the lignin samples to adsorb the dye (the drop-in adsorption capacity was 100-fold, from 42 to 0.45 mg g^–1^). This indicated and was confirmed by XPS that π–π interactions play a negligible role (<1% of total bonding) in adsorption. It was found that the carbonyl functional groups of lignin do not contribute to the adsorption of N-based organic cations Despite the high activity of −COOH groups, they contribute 26.7 and 15.8 folds less than aliphatic (including benzylic) and phenolic −OH, correspondently. This is attributed to the lower abundance of these functional groups in the lignin, present in the following ratios: 1(−COOH):7.5(aliphaticOH):10.7(phenolicOH). The aliphatic and phenolic hydroxyls are crucial for the adsorption performance but adsorb through different interactions. The aliphatic −OHs are important for H-bonding, while phenolic −OH, together with −COOH, is responsible for electrostatic attractions. Specific blocking of aliphatic and phenolic −OH in lignin samples showed that 61% of the adsorption of MB^+^ on Indulin-AT occurs due to H-bonding and 38% occurs through electrostatic interactions.

This study not only elucidates the fundamental mechanism of MB adsorption to lignin but also provides a pathway by which further potential modifications can improve the performance of lignin-based adsorbents. Future work will focus on adsorbing other organic molecules, investigating the adsorption of nonplanar structures to estimate the influence of sterics, as well as adsorbing anionic species capable of H-bond.

## Supporting Information Available

Details about quantitative functional group content in kraft lignin Indulin AT and modified derivatives, FTIR bands observed in kraft lignin Indulin AT, and modified derivatives before and after adsorption of Methylene Blue (PDF)
