# Supplementary material for: Unveiling the Nature of lignin’s Interaction with Molecules: A Mechanistic Understanding of Adsorption of Methylene Blue Dye
Source: Biomacromolecules. 2024 Jun 17;25(7):4292–304. doi: 10.1021/acs.biomac.4c00371 (PMC11238330; doi:10.1021/acs.biomac.4c00371)
Supplement: Supplementary file 1 — bm4c00371_si_001.pdf [file bm4c00371_si_001.pdf]

Supporting Information

Unveiling the nature of lignin's interaction with molecules: a mechanistic understanding of adsorption of Methylene Blue dye

Oleg Tkachenko[1], Daryna Diment[2], Davide Rigo[2], Maria Strømme[1], Tetyana M. Budnyak[1,3,4]*

[1]*Division of Nanotechnology and Functional Materials, Department of Materials Science and Engineering, Uppsala University, Sweden;*
[2]*Department of Bioproducts and Biosystems, Aalto University, Finland;*
[3]*Department of Earth Sciences, Uppsala University, Sweden*;
[4]*Wallenberg Initiative Materials Science for Sustainability, Department of Earth Sciences, Uppsala University, Sweden*

Corresponding Authors: Tetyana.Budnyak@angstrom.uu.se; Tetyana.budnyak@geo.uu.se

Number of Pages: 3

Number of Figures: 1

Number of Tables: 1

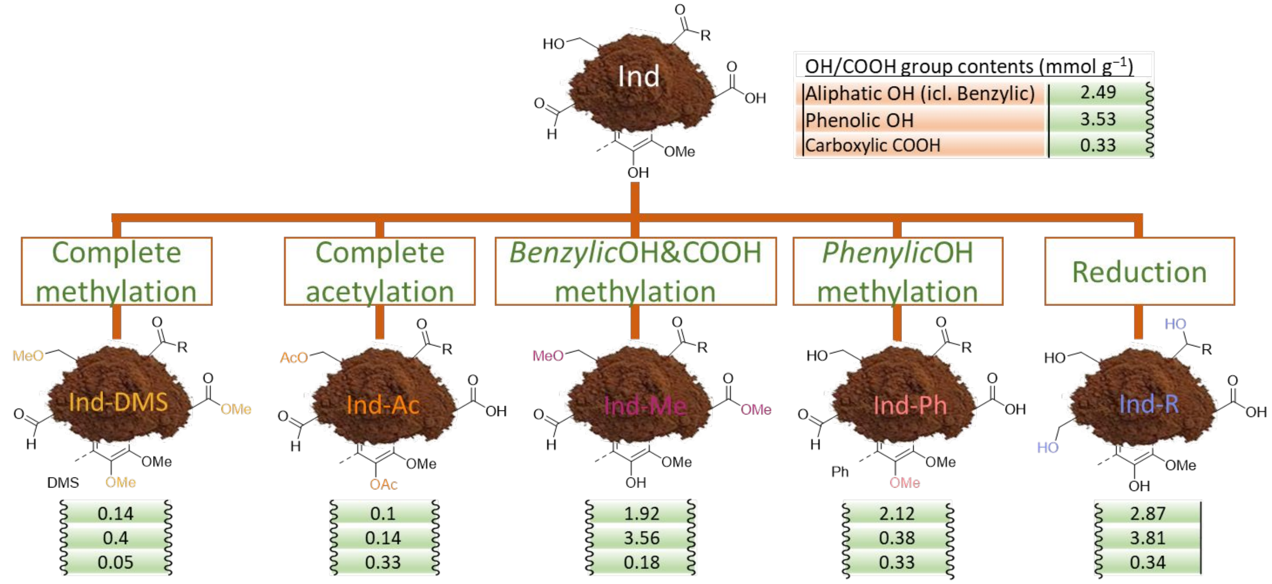

Figure S1. Scheme of modified lignin fabrication and their functional group content. More details in our previous work. [Diment, D.; Tkachenko, O.; Schlee, P.; Kohlhuber, N.; Potthast, A.; Budnyak, T. M.; Rigo, D.; Balakshin, M. Study toward a More Reliable Approach to Elucidate the Lignin Structure–Property–Performance Correlation. *Biomacromolecules* **2024**, *25* (1), 200–212. https://doi.org/10.1021/acs.biomac.3c00906.].

Table S2. The FTIR bands observed in *Ind*, *Ind-R*, *Ind-Me*, and *Ind-Ph* before and after the adsorption of MB

	Ind	*Ind-R*	*Ind-Me*	*Ind-Ph*	*Ind/ MB-1*	*Ind-R/ MB-2*	*Ind-R/ MB-3*	*Ind-Ph/ MB-4*	*Ind-Me/ MB-5*
Regions 1a and 1b									
Asymmetrical and symmetrical C–H stretch in CH_3 and CH_2 groups	2935(*m*) 2839(*m*)	2937(*m*) 2838(*m*)	2939(*m*) 2838(*m*)	2935(*m*) 2835(*m*)	2934(*m*) 2839(*m*)	2931(*m*) 2840(*m*)	2931(*m*) 2840(*m*)	2934(*m*) 2839(*m*)	2939(*m*) 2835(*m*)
C-H out-of-plane deformation vibrations in positions 2, 5, and 6 of G lignin units	854(*m*) and 814(*m*)	853(*m*) and 812(*m*)	854(*m*) and 815(*m*)	853(*m*) and 808(*m*)	855(*m*) and 816(*m*)	853(*m*) and 811(*m*)	854(*m*) and 810(*m*)	852(*m*) and 808(*m*)	854(*m*) and 815(*m*)
C–H out-of-plane bending vibrations of MB aromatic ring	-	-	-	-	883(*m*)	883(*m*)	883(*m*)	883(*w*)	883(*m*)
Regions 2									
O-H bending in phenolic OH	1367(*w*)	1365(*w*)	1364(*w*)	1361(*vw*)	1385(*w*)	1385(*m*)	1385(*m*)	1385(*vw*)	1383(*w*)
$n_{het}(C{=}S^+)$	-	-	-	-	1351(*w*)	1351(*w*)	1351(*w*)	1351(*vw*)	1351(*w*)
Stretching n(C-N) in N-CH_3	-	-	-	-	1327(*m*)	1327(*m*)	1327(*m*)	1327(*w*)	1327(*m*)
Regions 3									
n(-N-$(CH_3)_2$…H bond	-	-	-	-	2713(*w*)	-	2713(*w*)	-	-
Regions 4									
Aromatic C-H in-plane deformation	1125-1140(*s*)	1125-1139(*s*)	1124-1141(*w*)	1138(*s*)	1124-1136(*s*)	1125-1135(*s*)	1125-1136(*s*)	1138(*s*)	1138(*s*)
d_{het}(CH)	-	-	-	-	1172*(vw)*	-	1172*(vw)*	-	-
Regions 5									
C=O stretch, which are parts of conjugated p-substituted aryl ketones, and esters groups	1765-1636(*b*)	-	1750-1680(*b*)	1760-1650(*b*)	1765-1636(*b*)	-	**-**	1760-1650(*b*)	1750-1680(*b*)
C=O stretch in unconjugated ketones, carbonyls and in ester groups	-	1708(*m*)	-	-	-	1708(*m*)	1708(*m*)	-	-
Aromatic skeletal vibrations; C=O stretch; n_{het}(C-N) and n_{het}(C-C) for samples with MB	1593(*m*)	1596(*m*)	1597(*m*)	1585(*m*)	1595(*s*)	1595(*m*)	1595(*m*)	1587(*m*)	1597(*m*)
Regions 6									
-HC=CH- out-of-plane deformations	~968(*w*)	~969(*w*)	969(*w*)	966(*vw*)	~968(*w*)	~968(*w*)	~969(*w*)	966(*vw*)	968(*w*)
C-H out-of-plane (aromatic) deformations	~923(*w*)	~923(*w*)	922(*vw*)	-	~923(*w*)	~923(*w*)	~923(*w*)	-	922(*vw*)
N_{het}-HO deformations	-	-	-	-		930-945		-	-

vw – very weak, *w* – weak, m – medium, b – broad, s – strong
